# Paradoxical leadership, team adaptation and team performance: The mediating role of inclusive climate

**DOI:** 10.3389/fpsyg.2023.1052732

**Published:** 2023-04-06

**Authors:** Weixuan Meng, Zhihao Xu, Zulayati Abuliezi, Yaohui Lyu, Qi Zhang

**Affiliations:** ^1^Shanghai Key Laboratory of Mental Health and Psychological Crisis Intervention, School of Psychology and Cognitive Science, East China Normal University, Shanghai, China; ^2^Institute of Brain and Education Innovation, East China Normal University, Shanghai, China; ^3^School of Economics & Management, Tongji University, Shanghai, China

**Keywords:** paradoxical leadership, inclusive climate, team adaptation, team performance, trickle-down effect

## Abstract

In an increasingly complex and changing competitive environment, organizations inevitably face various conflicting demands, such as the contradiction between the psychological needs of employees and the organization’s performance requirements. Paradoxical leadership could focus on these competing needs of the organization and employees in multiple ways simultaneously. According to the trickle-down effect of social learning theory, we investigated whether and how paradoxical leadership may increase team adaptation and team performance. The study had a time-lagged survey design and included 254 team members and 60 leaders in 60 work teams in mainland China. The results of the structural equation modeling analysis indicated that paradoxical leadership is an essential predictor of team adaptation and performance, and that inclusive climate is mediating in this relationship. Our findings reveal a mechanism underlying the benefits of paradoxical leadership on team adaptation and team performance from a team-level perspective.

## 1. Introduction

In the face of a constantly developing competitive environment, the contradiction of demands within an organization is becoming increasingly prominent and permanent (Lewis, 2000). Many factors contribute to these contradictory demands, such as an emphasis on both short-term profits and long-term development, a desire to maintain both stability and flexibility, and the expectation that employees work independently while also strengthening teamwork. Zhang et al. (2015) argued that paradoxical leadership could simultaneously focus on the competing demands of the organization and subordinates in multiple ways (Julmi, 2021).

Paradoxical leadership adopts behaviors that appear to be inconsistent but are interrelated. For example, paradoxical leaders protect the interests of organizations by being self-centered, maintaining distance and employee homogeneity, enforcing high standards, and making final decisions to improve performance (Zhang and Han, 2019; Zhang and Liu, 2022). Meanwhile, paradoxical leaders also consider the individual needs of their employees by being other-centered, keeping intimate and personal, and allowing flexibility and autonomy (Zhang et al., 2015; Zhang and Liu, 2022).

Prior studies have shown that paradoxical leadership has positive correlations with employee voice behavior (Lu, 2020), followership behavior (Jia et al., 2018), proactive behavior (Peng and Li, 2018), ambidexterity (Zhang et al., 2016; Wang, 2018), and job performance (She and Li, 2017; She et al., 2020). A longitudinal study found that paradoxical leadership was significantly and positively related to team knowledge innovation. In this relationship, knowledge creation and integration are critical mediating variables (Luo et al., 2015). However, research on the impact of paradoxical leadership on teams has a single perspective that mainly focuses on innovation (Luo et al., 2015, 2017; Dashuai and Bin, 2020; Zhang et al., 2022) and less on other team outcomes, in particular, team adaptation and team performance. Although, many studies have found that paradoxical leadership positively predicts employee performance (e.g., Zhang et al., 2021), team performance is not an aggregation of individual employee performance but one of the most critical indicators of team effectiveness, including interaction and coordination (Yang et al., 2019).

Moreover, these studies focused on the single impact of paradoxical leadership, ignoring its “both … and …” characteristics of specific influences. Paradoxical leaders, for example, could maintain the unity between emotional connection and control with their subordinates, a beneficial intermediate state (Zhang et al., 2015, 2022). This allows subordinates with a high degree of autonomy to meet the conditions required for corporate innovation (Yi et al., 2019; Dashuai and Bin, 2020). With a management style that unites control and empowerment, the paradoxical leader could balance team adaptation and performance.

The present study explores the two impacts of paradoxical leadership at the team level based on the trickle-down effect of Social Learning Theory. We believe that paradoxical leadership could improve both team adaptation and team performance. This means that teams could be flexible while accomplishing performance requirements.

## 2. Theoretical background and hypotheses

### 2.1. Trickle-down effect and social learning theory

The trickle-down effect in organizations has received increasing attention (Venkataramani et al., 2010; Draconi and Kuenzi, 2012; Ambrose et al., 2013). It refers to leader-subordinate interactions in which leaders display specific traits and behaviors that influence similar traits and behaviors of their team members (Aryee et al., 2007). For example, leaders transmit ethical leadership behaviors to subordinates. Ruiz et al. (2011) found that leaders’ ethical behaviors influence employees’ civic organizational behavior, job satisfaction, and willingness to leave. The leader is probably the essential reference for subordinates. When leaders display an ethical image, it gives staff a sense of being cared for and trusted. Thus, it promotes work outcomes.

Weiss (1977, 1978) introduced social learning theory to study managerial organizations. According to this theory, leaders are role models for team members. Team members demonstrate certain attitudes and behaviors by imitating those who have trusted role models (Bandura, 1986). This is one of the leading frameworks to explain the trickle-down effect (Wang et al., 2015). The more subordinates perceive the leader as having status and success, the more they imitate and learn from the leader (Weiss, 1977). For instance, some scholars have argued that the “tone at the top” is critical to team climate. Therefore,the climate and principles created by senior managers can substantially impact team members behavior (Barney, 2005).

In some studies on leadership, researchers have found that leaders have a trickle-down effect on team members (Mayer et al., 2009; Liu et al., 2012; Mawritz et al., 2012). When the leader-follower relationship is positive, lower-level employees improve their work response through the trickle-down effect of the ethical norms of the higher-level leader (Mayer et al., 2009). In addition, there is a learning phenomenon in the leader-team. For example, Tucker et al. (2016) explored a transfer effect on safety based on social learning theory. Leaders’ attention to psychological safety issues creates a safe climate and culture for the organization and team. This eventually influences employees’ attitudes and behaviors. A study based on rooting theory found that paradoxical leader’s help subordinates learn to reduce stress and anxiety and cope with tension, thus developing paradoxical mindsets (Yin, 2022). Therefore, this study argues that when leaders commit paradoxical behaviors, it gives the followers a sense of inclusion and therefore learn from it. This builds an inclusive team climate and good team performance eventually.

### 2.2. Paradoxical leadership, team adaptation, and team performance

Teams are an integral part of organizations, and an effective team can adapt to various situations (Burke et al., 2006b). In organizations, team adaptation is as important as individual adaptation. Team adaptation refers to teams’ judgments regarding urgent environmental changes and the consequences of such adjustments (Burke et al., 2006b; Baard et al., 2014). Research has shown that leadership types impact team member adaptation. For example, transactional leadership enhances positive employee emotions, thus facilitating team adaptation in crisis scenarios (Sommer et al., 2016). In addition, team performance is the outcome of a team achieving predetermined goals (Hackman, 1987; Sundstrom et al., 1990). Team performance is still essential in examining team effectiveness (Yang et al., 2019). This study explores the effects of paradoxical leadership on team adaptation and performance based on the trickle-down effect of social learning theory.

Furthermore, as previously mentioned, leaders’ behaviors and attitudes would filter downward, and team members will imitate their behaviors and attitudes and thus share similarities with their style. Kim et al. (2020) found a trickle-down effect of abusive management based on social learning theory. The findings revealed that abusive supervisory behavior is trickle-down and is associated with emotional exhaustion. The positive association between abusive supervisory behavior and emotional exhaustion is higher when supervisors’ task performance is higher. Another study confirmed that alternative learning and organizational inclusive climate jointly mediated the relationship between the inclusive leadership of top managers and that of supervisors (Zhong et al., 2022).

On the one hand, paradoxical leaders show more flexibility in their work, and this behavior trickles down, so the whole team has a higher degree of flexibility in dealing with problems (Mammassis and Schmid, 2018). On the other hand, paradoxical leadership allows subordinates to keep their individuality and gives autonomy, contributing to team adaptability (Rico et al., 2022). Furthermore, in a study of ethical leadership and trickle-down effects, it was found that when leaders maintain good ethics, ethical behavior trickles down from higher to lower levels, resulting in improved team members’ work outcomes; and that this approach improves the performance of the “leader-follower” relationship (Ruiz et al., 2011). However, paradoxical leaders also give employees flexibility and autonomy while maintaining control over their decisions and enforcing strict job requirements. Based on trickle-down effect of social learning theory, the leader’s strict control over various boundaries at work would trickle down to the team. Then team members would observe and imitate the leader’s behavior and attitudes, thus ensuring that team performance remains at a reasonable level. Therefore, we propose the hypothesis that:

*Hypothesis 1:* Paradoxical leadership has a positive effect on team adaptation.

*Hypothesis 2:* Paradoxical leadership has a positive effect on team performance.

### 2.3. The mediating role of inclusive climate

Past research has noted that team leaders are critical in creating an inclusive organizational culture (Wasserman et al., 2008; Shore et al., 2011). An inclusive climate refers to employees’ shared beliefs about achieving fair employment practices, integration of differences, and inclusiveness in decision-making (Nishii, 2013). In a highly inclusive climate, employees are seen as insiders and have a sense of belonging. Employees’ uniqueness is highly valued, and they are encouraged to maintain their uniqueness and contribute fully to the collective (Ferdman, 2017).

According to social learning theory, team members learn the behaviors and attitudes of leaders in work scenarios, which in turn co-construct the appropriate team climate. Liden et al. (2014) sampled 961 employees from 71 restaurant chains. They showed that servant leadership behaviors of managers could help employees build a servant culture through the trickle-down effect. Paradoxical leaders are both self-centered and other-centered, which allows them to maintain their core influence, demonstrate assertive and exemplary leadership, and allow for individualization so that subordinates feel respected, affirmed, and supported (Jin, 2017). Moreover, they would give subordinates autonomy in their work, which would cause subordinates to identify and imitate, thus building a safe and inclusive team atmosphere to better utilize their influence and initiative (Zhang et al., 2015; She and Li, 2017; Peng and Ma, 2018).

Team climate is a shared perception by team members of the team’s work environment (Anderson and West, 1994). In an inclusive team climate, team members could reduce mutual blame during problem feedback analysis while increasing objective and accurate assessment of the response process, leading to team innovation and successful team adaptation to future changes (Burke et al., 2006b). Moreover, previous research has found that perceived team inclusiveness positively impacts relationship performance, team decision quality (Bosselaar, 2015), and team performance (Fang, 2014; Jansen et al., 2014). Also, inclusive leadership can facilitate team communication and knowledge sharing and positively affect team performance and employee innovation performance (Zhong et al., 2018).

In summary, paradoxical leadership leads to an inclusive team climate through a trickle-down effect. This climate enables team members with different backgrounds and values to respect each other and encourages their active participation in decision-making (Nishii, 2013). In addition, an inclusive climate eliminates concerns about “making mistakes/failure” while encouraging the expression of different perspectives (Tang and Zhang, 2015). In turn, team members share different perspectives, information, and knowledge (Zhong et al., 2018; Xu and Zhang, 2019), ultimately enhancing team adaptation and performance. Therefore, we propose that:

*Hypothesis 3:* Inclusive climate would mediate the effect of paradoxical leadership on team adaptation.

*Hypothesis 4:* Inclusive climate would mediate the effect of paradoxical leadership on team performance.

## 3. Method

### 3.1. Participants and procedure

This study used convenience sampling. Data for this study were collected in two waves with an interval of one month from several enterprises in mainland China. We distributed questionnaires based on teams *via* an online survey platform (Wen Juan Xing, https://www.wjx.cn). A team consists of a team leader and at least three team members who have worked with each other on the team for at least one year. At Time 1, all the participants provided demographic information. In addition, team members reported the leader’s paradoxical leadership and team-inclusive climate. At Time 2, team members reported team adaptation while team leaders evaluated team performance. Participants who completed both surveys were paid 30 CNY (roughly 4.33 USD).

After excluding invalid data (missing data or repeated answers), the final valid sample consisted of 254 team members nested in 60 teams (usable data rate = 82.70%). The size of the teams ranged from 3 to 10 team members, with an average of 5.39 (SD = 2.18). Of the team members, 50% were males, averaging 34.62 years (SD = 6.30). The average job tenure was 9.40 years (SD = 6.56). Of the team leaders, 41.70% were male, and the average age was 34.10 years (SD = 4.88). The average tenure for team leaders was 9.85 years (SD = 5.11).

### 3.2. Measures

We used scales from the existing literature to measure the variables. Scales in English were translated into Chinese following standard translation and back-translation procedures (Brislin, 1980).

#### 3.2.1. Paradoxical leadership

We used the 22-item scale developed by Zhang et al. (2015) to measure paradoxical leadership behavior (i.e., “My leader maintains overall control but gives subordinates appropriate autonomy”). The scale contains five dimensions: (1) combining self-centeredness with other-centeredness (5 items); (2) treating subordinates uniformly while allowing individualization (5 items); (3) enforcing work requirements while allowing flexibility (4 items); (4) maintaining both distance and closeness (4 items); and (5) maintaining decision control while allowing autonomy (4 items). Team members rated their agreement with the items on a five-point Likert scale ranging from 1 (*strongly disagree*) to 5 (*strongly agree*). The Cronbach’s alpha was 0.94 in this study.

#### 3.2.2. Team adaptation

Team adaptation was measured by three items (i.e., “After agreements have been made in this team, everyone does things in the same manner”) (Wiedow and Konradt, 2010). Team members rated their agreement with the items on a five-point Likert scale ranging from 1 (*never*) to 5 (*always*). The Cronbach’s alpha was 0.95 in this study.

#### 3.2.3. Team performance

Team leaders assessed team performance through six items (i.e., “Team members work effectively”) (Tjosvold et al., 2006). Response categories ranged from “1 = *strongly disagree*” to “5 = *strongly agree*.” The Cronbach’s alpha for this 6-item team performance scale was 0.95 in this study.

#### 3.2.4. Inclusive climate

We measured inclusive climate with a 15-item scale (*α* = 0.95) developed by Nishii (2013) and translated into Chinese by Xu and Zhang (2019). For example, “This company (or department) has a fair promotion process.” Team members rated their agreement with the items on a five-point Likert scale ranging from 1 (*strongly disagree*) to 5 (*strongly agree*). The Cronbach’s alpha was 0.96 in this study.

#### 3.2.5. Control variables

We controlled the effects of two team characteristics variables (team leader tenure and team size) on team adaptation and team performance. Previous research suggests that leaders with longer tenure and team size may be more likely to lead to better team adaptation (Finkelstein and Hambrick, 1990; Christian et al., 2017).

### 3.3. Data analysis

We conducted a confirmatory factor analysis (CFA) in Mplus 7.0. In addition, we used IBM SPSS 23.0 to conduct descriptive statistics and correlation analyses. Lastly, we performed a Structure Equation Model (SEM) to test the hypotheses using Mplus 7.0.

## 4. Results

### 4.1. Data aggregation

The analysis in this study was conducted at team-level, where team performance is team-level data. However, evaluations of paradoxical leadership, inclusive climate, and team adaptation were obtained at the individual level. Therefore, individual-level data needed to be aggregated at the team-level. We calculated intra-class correlation (ICC), within-group agreement index *R_wg_*, and one-way analyses of variance (ANOVA) *F*-values to test the appropriateness of aggregation at the team level (Bliese, 2000). ICC indicates whether variables are rated consistently enough within teams to justify aggregation at the team level (Bliese, 2000). *R_wg_* evaluates the consistency of the team members’ ratings, which ranges from 0 to 1, indicating complete disagreement to the agreement among team members. Values of 0.70 or above are adequate (George, 1990; George and Bettenhausen, 1990; Gevers et al., 2020). The results showed that ICC values ranged from 0.27 to 0.37, the average *R_wg_* ranged from 0.93 to 0.98, and the one-way ANOVA F-values ranged from 2.53 to 3.40 (*p* < 0.001), suggesting the appropriateness of aggregation (see Table 2; James et al., 1984; LeBreton and Senter, 2008).

### 4.2. Confirmatory factor analysis

The discriminant validity of study variables was tested by confirmatory factor analysis. The results showed that the four-factor model fit the data better than any of the alternative models (CFI = 0.96, TLI = 0.95, RMSEA = 0.09, SRMR = 0.07, *χ^2^/df* = 1.55, *p* < 0.001) (Bentler and Bonett, 1980; Cheung and Rensvold, 2002). These results suggest that common method bias is not significant in our study (Podsakoff et al., 2003). Given the results, all constructs were applied in the following analyses (see Table 1).

**Table 1:** Discriminant validity analysis.

Model	*χ* ^2^	*df*	CFI	TLI	RMSEA	SRMR
Four-factor model	151.67	98	0.96	0.95	0.09	0.07
Three-factor model PL + IC, TA, TP	413.02	101	0.74	0.69	0.23	0.21
Three-factor model PL, IC, TA + TP	209.88	101	0.91	0.89	0.13	0.07
Two-factor model PL + IC, TA + TP	753.13	108	0.47	0.41	0.32	0.43
One-factor model	431.93	104	0.73	0.69	0.23	0.14

CFI, comparative fit index; TLI, Tucker–Lewis index; RMSEA, root mean square error of approximation; SRMR, standardized root mean square residual. PL, paradoxical leadership; IC, inclusive climate; TA, team adaptation; TP, team performance.

### 4.3. Descriptive statistics and correlation analysis

Descriptive data are shown in Table 2. There were significant positive correlations between paradoxical leadership, inclusive climate, team adaptation, and team performance (*ps* < 0.01).

**Table 2:** Descriptive statistics, aggregation indices, and correlations for study variables.

Variables	*M*	SD	ICC	*R_wg_*	1	2	3	4	5	6
1. Team leader tenure	9.56	5.16			1					
2. Team size	5.88	2.17			0.08	1				
3. Paradoxical leadership	3.94	0.35	0.27	0.98	0.16^*^	0.01	1			
4. Inclusive climate	4.05	0.48	0.37	0.97	−0.02	0.10	0.52^**^	1		
5. Team adaptation	4.22	0.46	0.31	0.93	−0.06	0.15^*^	0.50^**^	0.85^**^	1	
6. Team performance	3.91	0.81			0.22^**^	0.23^**^	0.40^**^	0.36^**^	0.30^**^	1

*N* = 60; SD, standard deviation; ^*^p < 0.05, ^**^p < 0.01, ^***^p < 0.001.

### 4.4. Testing of hypotheses

A structural equation model (SEM) performed by Mplus7.0 was used to test ourhypotheses.While controlling for leadership tenure and team size, paradoxical leadership has a significant positive effect on team adaptation (total effect = 0.51, *p* < 0.001, 95% *CI* = [0.28, 0.72]) and team performance (total effect = 0.65, *p* = 0.02, 95% *CI* = [0.11, 1.03]), supporting Hypothesis 1 and 2.

**Figure 1:**
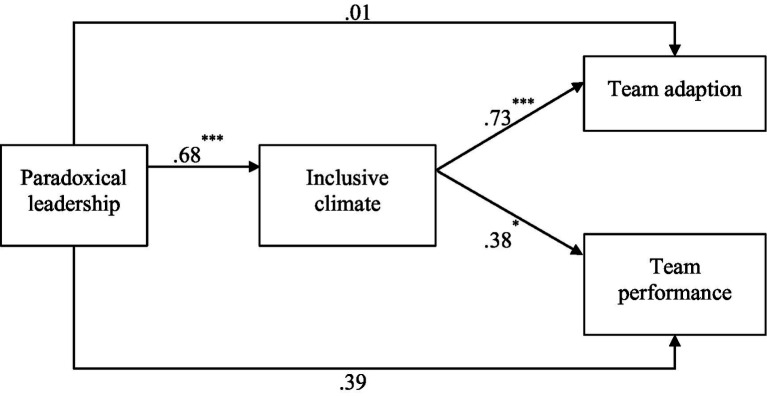
Structural equation model. ^*^p < 0.05, ^**^p < 0.01, ^***^p < 0.001.

Figure 1 demonstrates that the path coefficient from paradoxical leadership to inclusive climate was significant [*β =* 0.68, *p* < 0.001, 95% *CI* = (0.50, 0.87)]. There was a significant positive path coefficient from inclusive climate to team adaptation [*β* = 0.73, *p* < 0.001, 95% *CI* = (0.60, 0.86)]. Finally, the path coefficient from inclusive climate to team performance was 0.38 [*p* = 0.04, 95% *CI* = (0.10, 0.68)].

Table 3 demonstrates that inclusive climate significantly mediated the relationship between paradoxical leadership and team adaptation [indirect effect = 0.50, *p* < 0.001, 95% *CI* = (0.36, 0.68)] in support of Hypothesis 3. Similarly, inclusive climate significantly mediated the relationship between paradoxical leadership and team performance (indirect effect = 0.26, *p* = 0.07, 95% *CI* = [0.07, 0.52]), supporting Hypothesis 4.

**Table 3:** Model tests.

Path	Effect	Standard error	95% Confidence interval	
			Lower limit	Upper limit
Paradoxical leadership-inclusive climate-team adaptation				
Total effect	0.51	0.17	0.28	0.72
Indirect effect	0.50	0.10	0.36	0.68
Direct effect	0.01	0.11	−0.13	0.16
Paradoxical leadership-inclusive climate-team performance				
Total effect	0.65	0.28	0.11	1.03
Indirect effect	0.26	0.14	0.07	0.52
Direct effect	0.39	0.27	−0.10	0.79

## 5. Discussion

The current study investigated the relationship between paradoxical leadership, team adaptation, and team performance using the trickle-down effect (Venkataramani et al., 2010; Draconi and Kuenzi, 2012; Ambrose et al., 2013) of social learning theory (Weiss, 1977, 1978) as the theoretical framework and examined the mediating role of inclusive climate in relationships. The results showed that paradoxical leadership has a significant positive relationship with inclusive climate, team adaptation, and team performance, while an inclusive climate has a significant positive relationship with team adaptation and team performance.

Our findings thus support the notion that team leaders are critical in fostering an inclusive team climate and enhancing team adaptation and team performance (Marks et al., 2000; Wasserman et al., 2008; Shore et al., 2011; Li et al., 2018). The dialectical thinking and integrated perspective of paradoxical leadership facilitate a more systematic and comprehensive view of environmental change (Lewis and Smith, 2014), setting the groundwork for a series of adaptive plans, programs, and the effectiveness of mission execution (Waller et al., 2004; Burke et al., 2006a). These characteristics of paradoxical leaders contribute to an inclusive team climate, team adaptation, and team performance (Lewis and Smith, 2014; Zhang et al., 2015; Waldman and Bowen, 2016).

In the present study, an inclusive climate had a significant positive relationship with team adaptation and team performance. An inclusive climate could facilitate team communication, contributing to team resource integration, information, and knowledge sharing (Ely and Thomas, 2001; Zhong et al., 2018). Prior studies have also demonstrated that an inclusive climate facilitates task coordination in unusual situations (Le, 2016), enabling team members to readily alter behaviors in response to changing, leading to improved team adaptation and team performance. Furthermore, an inclusive team atmosphere permits employees to make mistakes, allowing trial and error, ultimately leading to better team adaptation and team performance (Le, 2016). The current results are, therefore, consistent with prior literature (Cho and Barak, 2008; Jansen et al., 2014; Bosselaar, 2015).

Lastly, we found that an inclusive climate mediates the relationship between paradoxical leadership and team adaptation; and the relationship between paradoxical leadership and team performance. The behavior style of paradoxical leadership enables team members to feel respected and affirmed (Zhang et al., 2015). The varied opinions of team members are accepted and valued, and each individual’s uniqueness is appreciated in an environment of good communication (She and Li, 2017; Peng and Ma, 2018), thus contributing to an inclusive climate of equitable employment practices, integration of differences, and inclusion in decision-making (Nishii, 2013). These characteristics, in turn, enhance team adaptation and team performance.

### 5.1. Theoretical implications

The present study deepens our understanding of the effectiveness of the emerging style of paradoxical leadership based on the perspective of the trickle-down effect of social learning theory. This study focused on the characteristics of paradoxical leaders “both … and ….”. Through a management style that combines control and empowerment, paradoxical leaders could maintain a balance between team adaptation and team performance. For one thing, paradoxical leaders may show more flexibility in their work, and this behavior trickles down, allowing the entire team to have a higher level of flexibility in dealing with problems (Mammassis and Schmid, 2018). At the same time, paradoxical leadership allows employees to maintain individuality and give autonomy, contributing to team adaptation (Rico et al., 2022). For another, paradoxical leaders give subordinates flexibility and autonomy while maintaining control over their decisions and strict enforcement of work requirements. The leader’s strict control over workplace boundaries trickles down to the team, and team members observe and imitate the leader’s behavior and attitudes, thereby contributing to team performance (Ruiz et al., 2011). The findings indicate that paradoxical leadership has a positive impact on team adaptation and team performance.

Second, the present study explored mechanisms involved in the effect of paradoxical leadership on team adaptation and team performance based on the trickle-down effect of social learning theory, with inclusive climate as a potential mediating factor (Wasserman et al., 2008; Shore et al., 2011). The results show that paradoxical leaders promote team adaptation and team performance by actively creating an inclusive climate. This result contributes to our understanding of how paradoxical leadership may impact team adaptation and team performance and provides a new perspective for future research. Meanwhile, it demonstrates the importance of creating an inclusive climate for team adaptation and team performance.

Finally, the results from the current study enrich the empirical research on paradoxical leadership by examining its effects at the team level. Previous research on paradoxical leadership has primarily explored effects at the individual level (Zhang et al., 2016; She and Li, 2017; Jia et al., 2018; Peng and Li, 2018; Wang, 2018; Lu, 2020; She et al., 2020), with some organization-level research (Wang et al., 2018) in the form of case studies. However, very little empirical research has previously focused on the team level (Luo et al., 2015; Zhang et al., 2016, 2022; Dashuai and Bin, 2020). The current study found a positive impact of paradoxical leadership on team adaptation and team performance at the team psychological level, with climate as an underlying mechanism for this effect.

### 5.2. Practical implications

The results from the reported study have practical implications for organizational management and leadership behavior in the workplace.

First, paradoxical leadership behaviors contribute to team adaptation and team performance, providing a new perspective for management practices. Organizations should take advantage of the paradoxical leader’s ability to think broadly. In contrast to the traditional leadership style of “one or the other” (Lewis, 2000; Smith and Lewis, 2011), paradoxical leadership adopts dialectical thinking when faced with dynamic changes and resolves dilemmas by maintaining a systematic and integrated perspective and constantly accepting, integrating, and coordinating old and new contradictions and conflicts, thus improving team adaptation and team performance (Lewis and Smith, 2014; Zhang et al., 2015; Waldman and Bowen, 2016; Zhang and Liu, 2022).

Second, organizations should exploit the paradoxical leader’s ability to respond appropriately to unexpected situations (Smith and Lewis, 2011). The paradoxical leader could influence teams to adapt quickly to complex and changing situations through high-performance expectations and management support (Kauppila and Tempelaar, 2016). Even in emergencies, paradoxical leaders might guide their teams to develop a realistic strategy and ensure strict implementation of planned goals (Burke et al., 2006a; Kauppila and Tempelaar, 2016). At the same time, paradoxical leaders could learn from the practical experience of managing unexpected situations, enabling the team to gain functional adaptations that further enhance team performance (Tripathi, 2017; Zhu et al., 2019).

Finally, our results indicate that paradoxical leaders enhance team adaptation and team performance through an inclusive climate. Therefore, organizations could benefit from focusing on the positive effects of inclusive climate on enhancing team adaptability. Companies should focus their efforts on two levels. First, organizations should change their management mindset, optimize human resource management, and foster an inclusive climate in hiring, information evaluation, and decision-making at the organizational level by setting relevant rules and regulations. Second, at the leadership level, leaders should create a team climate with fair competition, mutual respect, and inclusive decision-making. In addition, team information resource sharing and integration of diverse thinking and perspectives will foster team adaptation and team performance.

### 5.3. Limitations and directions for future research

While the current study offers empirical support for organizational initiatives, several limitations exist.

First, the present study focused on the mediating effect of inclusive climate on the relationship between paradoxical leadership and team adaptation, and the relationship between paradoxical leadership and team performance. However, the boundary conditions of paradoxical leadership were not considered. Individual characteristics (such as mental model, team orientation, and personality traits) (Burke et al., 2006b; Le, 2016), job design characteristics (self-management) (Burke et al., 2006b), and physical environmental factors (such as team task characteristics, environmental pressure, resources, and technology) (Hackman, 1987) should be explored in future research.

Second, other mediators, such as team efficacy and reflexivity, may contribute to the relationship between paradoxical leadership and team adaptation, and the relationship between paradoxical leadership and team performance. Teams with a high level of efficacy (Bandura, 1997) include members who hold firm beliefs about the team’s ability to complete assigned tasks and achieve team goals. Through their distinctive leadership style, team leaders positively impact team members, improving their confidence to complete tasks and achieve team goals (Chen et al., 2019). Paradoxical leaders that treat subordinates equally and encourage positive characteristics such as flexibility may help boost team efficacy, which may, in turn, enhance team adaptation and team performance. Team reflexivity, the extent to which teams jointly reflect on and adapt their working methods and functioning (Schippers et al., 2015), may mediate between paradoxical leadership and team adaptation and team performance. Team reflectivity emphasizes an environment where team members communicate openly, learn from one another, and interact. Paradoxical leadership fosters such an environment, encouraging team members to collaborate to perform better (Yuan, 2019).

Lastly, although we divided two-time points to measure the main variables, the 1-month interval between the independent and dependent variables is too short for this study to remain essentially a cross-sectional study to make inferences about cause and effect. Future studies may consider longitudinal studies or quasi-experimental designs.

## Data availability statement

The raw data supporting the conclusions of this article will be made available by the authors, without undue reservation.

## Ethics statement

This study was carried out in accordance with the recommendations of the World Medical Association’s Declaration of Helsinki. The Ethics Committee of East China Normal University (code: HR2-1110-2020) approved this study. All participants were informed about the details of the study and gave their informed consent before participating. The patients/participants provided their written informed consent to participate in this study.

## Author contributions

WM and ZX: conceptualization. WM, ZX, and YL: data curation. ZA: formal analysis. QZ and YL: funding acquisition. ZX and YL: methodology. QZ and YL: project administration. WM and ZX: writing—original draft. ZA, YL, and QZ: writing—review and editing. All authors contributed to the article and approved the submitted version.

## Conflict of interest

The authors declare that the research was conducted without any commercial or financial relationships that could be construed as a potential conflict of interest.

## Publisher’s note

All claims expressed in this article are solely those of the authors and do not necessarily represent those of their affiliated organizations, or those of the publisher, the editors and the reviewers. Any product that may be evaluated in this article, or claim that may be made by its manufacturer, is not guaranteed or endorsed by the publisher.
